# Fabricating Surface-Functionalized CsPbBr_3_/Cs_4_PbBr_6_ Nanosheets for Visible-Light Photocatalytic Oxidation of Styrene

**DOI:** 10.3389/fchem.2020.00130

**Published:** 2020-03-10

**Authors:** Ping Qiu, Qiuhe Wang, Yizhou Zhao, Yi Dai, Yuanyuan Dong, Changli Chen, Qi Chen, Yujing Li

**Affiliations:** ^1^School of New Energy and Materials Science, China University of Petroleum, Beijing, China; ^2^Beijing Key Laboratory of Construction Tailorable Advanced Functional Materials and Green Applications, School of Materials Science and Engineering, Beijing Institute of Technology, Beijing, China

**Keywords:** halide perovskite, photocatalytic oxidation, surface functionalization, benzaldehyde, doping

## Abstract

The halide perovskite (PVSK) material, an excellent light absorber with fast carrier kinetics, has received increased attention as a potential photocatalyst for organic synthesis. Herein, we report a straightforward synthesis of chemically modified halide perovskite and its application as an efficient photocatalyst to convert styrene into benzaldehyde. A simple method is employed to synthesize the chemically modified CsPbBr_3_/Cs_4_PbBr_6_ nanosheets by using ZrCl_4_ to simultaneously achieve the Cl doping and the surface modification with Zr species. The photocatalytic oxidation rate of styrene to benzaldehyde catalyzed by surface-modified CsPbBr_3_/Cs_4_PbBr_6_ nanosheets under visible light can reach 1,098 μmol g^−1^ h^−1^, 2.9 times higher than that of pristine CsPbBr_3_/Cs_4_PbBr_6_ nanosheets (372 μmol g^−1^ h^−1^). The enhanced photocatalytic performance may originate from the modified band structure induced by the synergistic effect of Cl doping and surface modification, whereby the same methodology can be applied to MAPbBr_3_. This work demonstrates the surface modification of PVSK materials and their potential as efficient photocatalyst toward organic synthesis.

## Introduction

In recent years, halide perovskite (PVSK) material has received enormous attention due to its unique optoelectronic properties as light-absorbing material in solar cell devices. Based on their excellent photosensitivity, wide range of visible-light absorption (Yang et al., 2017), long carrier lifetime (Yang et al., 2015, 2016), and diffusion length (Xing et al., 2013; Dong et al., 2015), the halide perovskite material possesses the basic physicochemical properties of a good photocatalyst. Besides, the low cost, feasible processability, and adjustable energy levels of band edge make the PVSK a promising candidate for photocatalyst (Chen et al., 2015). However, the PVSK, when used alone, suffers from poor stability and difficulty in functionalization for photocatalysis. SnS_2_, Au, graphene oxide, and TiO_2_ have been employed to composite with PVSK as photocatalyst for organic synthesis and other reactions (Huang et al., 2018; Feng et al., 2019; Wang Q. et al., 2019; Wang X. et al., 2019), but very little effort has been made to address the surface of PVSK for efficient photocatalytic process.

Benzaldehyde plays a vital role in the industrial synthesis of numerous chemicals (Hu et al., 2016; Tong et al., 2016; Oliveira et al., 2017). The commonly used methods to synthesize benzaldehyde include the oxidation of benzyl alcohol, direct oxidation of toluene, hydrogenation of benzoic acid, indirect electrooxidation of toluene, and hydrolysis of benzyl chloride (Yadav and Haldavanekar, 1997; Lv et al., 2010; Nasrollahzadeh et al., 2016), which have problems such as complexity of processing, environmental footprint, high manufacturing cost, etc. Recently, the direct oxidation of styrene to benzaldehyde by environmentally friendly oxidants has attracted attention (Singh and Sinha, 2014; Fu et al., 2016; Narayanan et al., 2016). A number of heterogeneous catalysts have been used for the oxidation of styrene, including noble metal catalysts such as Au (Majeed et al., 2016; Wang et al., 2018), Ag (Gupta et al., 2017), Pt (Luo et al., 2015b), and Pd (Luo et al., 2015a), which have shown excellence in both activity and selectivity. However, high prices and finite reserves limit their practical application. Photocatalytic technique could be an optimistic approach to convert styrene based on non-precious metal with abundant reserves and low cost.

In this study, by employing the functionalized PVSK as the photocatalyst, we achieve the efficient conversion of styrene into benzaldehyde with visible light. We report a one-pot method based on the ligand-assisted precipitation (LARP) (Zhang et al., 2015; Zhu et al., 2019) to synthesize surface-modified CsPbBr_3_/Cs_4_PbBr_6_ nanosheets as a visible-light photocatalyst for the oxidation of styrene to benzaldehyde at room temperature using oxygen as the oxidant. We use ZrCl_4_ in modifying CsPbBr_3_/Cs_4_PbBr_6_ nanosheets to enhance the oxidation ability and promote the transfer of carriers. The chemical modification with ZrCl_4_ and the effect of surface functionalization on the properties of CsPbBr_3_/Cs_4_PbBr_6_ are investigated. In addition, the mechanism of the enhanced photocatalytic performance from ZrCl_4_-CsPbBr_3_/Cs_4_PbBr_6_ nanosheets toward the oxidation of styrene is reasonably proposed.

## Experimental

### Materials

Oleic acid (OAc, 90%, Sigma-Aldrich), n-octylamine (99.0%, Aladdin), N,N-dimethyl-formamide (DMF, 99.9%, Aladdin), cyclohexane (99.0%, Aladdin), methyl acetate (99.9%, Aladdin), n-Hexane (99.0%, Aladdin), styrene (99.9%, Aladdin), cesium bromide (CsBr, 99.9%, Sigma-Aldrich), cesium chloride (CsCl, 99.9%, Sigma-Aldrich), lead(II) bromide (PbBr_2_, 99.9%, Sigma-Aldrich), and zirconium (IV) chloride (ZrCl_4_, 99.9%, Sigma-Aldrich). All chemical reagents are used as received.

### Preparation: Synthesis of Surface-Modified Perovskite CsPbBr_3_/Cs_4_PbBr_6_ Nanosheets

**Figure 1:**
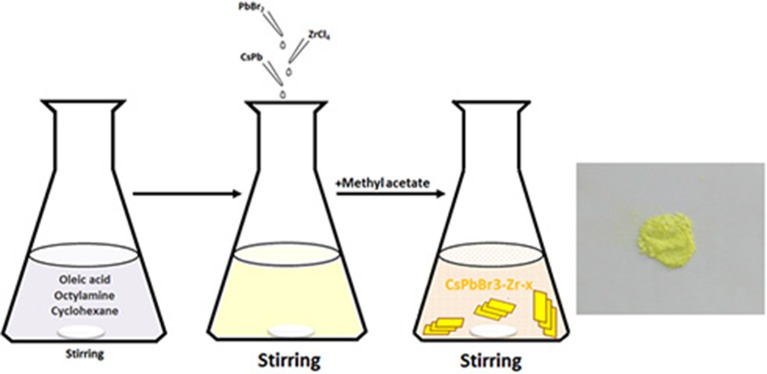
The schematic of the synthesis and photographs under visible light of ZrCl_4_-CsPbBr_3_/Cs_4_PbBr_6_ nanosheets.

The synthesis employs the ligand-assisted precipitation (LARP) method as illustrated in Figure 1. Three precursor solutions were prepared by dissolving 2.0 mmol CsBr in 1.0 ml of H_2_O, 2.0 mmol PbBr_2_ in 2.5 ml of DMF, and 0.25 mmol ZrCl_4_ in 0.2 ml of DMF, respectively. Then, 4 ml of oleic acid and 2 ml of n-octylamine were added to a vigorously stirred cyclohexane (200 ml). The PbBr_2_ solution, ZrCl_4_ solution, and CsBr solution were added. A microemulsion started to form and the solution turned from transparent into light yellow. Afterwards, methyl acetate (100 ml) was added to break up the emulsion. The as-synthesized CsPbBr_3_/Cs_4_PbBr_6_ nanosheets (named as CPB-Zr-0.25) were separated by centrifugation at 10,000 rpm for 10 min, and washed twice with toluene, dried at 80°C, and collected for storage. By changing the amount of ZrCl_4_ solution added, CPB-Zr-*x* (*x* = 0.25, 0.5, 0.75, 1) were obtained. The organic–inorganic hybrid lead halide perovskite materials MAPbBr_3_ and MAPbBr_3_-Zr-*x* nanosheets, named MAPB-Zr-*x* (MA = CH_3_${\text{NH}}_{3}^{+}$, *x* = 0.25, 0.5, 0.75, 1), were obtained with the same protocol by replacing CsBr with MABr. The unmodified CsPbBr_3_/Cs_4_PbBr_6_ nanosheets were synthesized with the same procedure except that the ZrCl_4_ precursor was not added. ZrO_*x*_Cl_*y*_ was prepared with the above procedure except that CsBr and PbBr_2_ were not added, as the comparison for photocatalytic evaluation.

### Photocatalytic Activity Measurements

Photocatalytic reactions were conducted in a 20-ml quartz flask matching the Pcx-50c multichannel photochemical reaction device with magnetic stirring at a rate of 500 rpm. The specific procedure was as follows: 20 mg of photocatalyst was dispersed into 9 ml of n-Hexane saturated with molecular oxygen and mixed with 1 ml of styrene. The photocatalytic reaction system was irradiated with a white light-emitting diode (LED) illumination (100 mW cm^−2^). After reaction for 5 h, the suspension was centrifuged at 10,000 rpm for 8 min and the supernatant was analyzed on Shimadzu GC-2014.

### Characterization

X-ray diffraction (XRD) analysis was performed on a Rigaku D/MAX2500 VB2+/PCX-ray diffractometer using Ni-filtered Cu Kα radiation (λ = 1.5406 Å) with a scanning angle (2θ) ranging 10°-60° and the scanning speed at 5°/min. UV-vis diffuse reflectance spectra (DRS) of the samples were studied by a UV-vis spectrophotometer (Perkin Elmer Lambda 950) from 300 to 800 nm. FEI Tecnai G2 F20 was used for high-angle annular dark-field scanning transmission electron microscopy (HAADF-STEM) imaging, and electron energy spectrum (EDX) is obtained from Super-EDX equipped on the TEM FEI G2 (60-300) TEM. The photoluminescence (PL) spectra were acquired by an FLS980 fluorescence spectrometer (steady state, lifetime, phosphorescence, Edinburgh Instruments Ltd.). The steady-state PL was irradiated with 370-nm excitation light. The time-resolved photoluminescence (TRPL) spectra were performed using a 375-nm laser emitter to determine the lifetime of photogenerated carriers. X-ray photoelectron spectroscopy (XPS) was used to study the chemical compositions and the chemical states by a Thermo escalab 250Xi photoelectron spectrometer. Ultraviolet photoelectron spectra (UPS) analysis was performed on British VG Scienta R4000, using He I line as ultraviolet light source with photon energy 21.2 eV, spectrum acquisition range (kinetic energy) 0–22 eV, and step length 0.02 eV. Surface photovoltage (SPV) spectra were conducted in a vacuum chamber with a quartz window. As-prepared samples were placed into a vacuum chamber and illuminated by monochromatic light from a 150-W Xe lamp filtered through an Oriel Cornerstone 130 monochromator (1–10 mW cm^−2^) within a range of 300–600 nm.

## Results and Discussions

### XRD, DRS, TRPL and SPV Spectra Analysis

**Figure 2:**
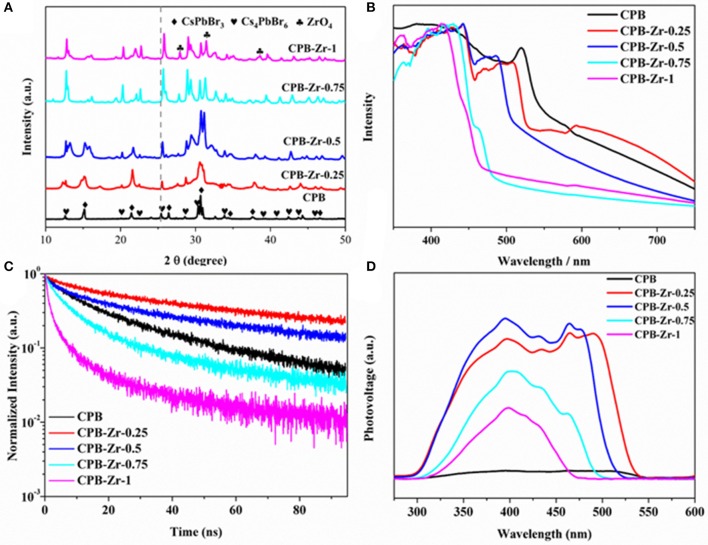
**(A)** XRD patterns, **(B)** UV-vis diffuse reflectance spectra, **(C)** TRPL spectra, and **(D)** SPV spectra of the samples: CPB and CPB-Zr-*x* (*x* = 0.25, 0.5, 0.75, 1).

Figure 2A shows the XRD pattern of CPB and CPB-Zr-*x* (*x* = 0.25, 0.5, 0.75, 1). The naked CPB shows a series of peak at 25.3°, 27.1°, 37.8°, and 48.0° (2θ), corresponding to the (001), (110), (002), and (220) crystallographic plane of CsPbBr_3_ (JCPDS card No. 18-0364), respectively, whereas the peak at 12.55°, 20.25°, 22.56°, and 25.55° (2θ) corresponds to the (012), (113), (300), and (024) crystallographic plane of Cs_4_PbBr_6_ (JCPDS card No. 73-2478), indicating that the CPB prepared by LARP method has a mixed structure of CsPbBr_3_ and Cs_4_PbBr_6_. With the increased amount of ZrCl_4_, the peak at peaks of CPB-Zr shifts to the higher values with decreased intensity of the diffraction peak. The positive shift of diffraction peaks may largely originate from the inclusion of Cl^−^ (from ZrCl_4_) into the lattice, whereby the Cl^−^ has a smaller radius than that of Br^−^. When the amount of ZrCl_4_ further increases, e.g., CPB-Zr-0.75 and CPB-Zr-1, new diffraction peaks start to appear. A thorough search of PDF database turns out that the newly appearing peaks are consistent with the characteristic diffraction peaks of zirconium oxide (JCPDS card No. 1-750). Figures S1a,b compare the core-level Cl 2p and Zr 3d XPS spectra from CPB and CPB-Zr-0.75. It can be clearly found the CPB-Zr-0.75 sample shows obvious Cl 2p and Zr 3d peaks, whereas the CPB shows no signal for either spectrum, confirming that the CPB-Zr-0.75 contains Zr and Cl. It can be referred that during the synthesis, part of Cl^−^ from ZrCl_4_ dopes into the CPB to replace Br^−^, while the rest forms ZrO_2_ on the surface of CsPbBr_3_/Cs_4_PbBr_6_ nanosheets (Xu et al., 2016).

The UV-vis diffuse reflectance spectra of as-prepared CPB and CPB-Zr-*x* (*x* = 0.25, 0.5, 0.75, 1) samples are shown in Figure 2B. The spectrum of CPB sample displays the absorption in the visible-light region exhibiting an absorption edge at 545 nm, while the CPB-Zr-*x* samples show blue shift of absorption edge. The ZrCl_4_ sample cannot absorb light as shown in Figure S2a. It is obvious that the chemical functionalization with ZrCl_4_ can significantly affect the optical absorption feature of CPB. By increasing the amount of ZrCl_4_, the absorption edge of the samples shows a further blue shift. In particular, the absorption edge of CPB-Zr-1 is at 473 nm. The optical bandgap value can be calculated via the Kubelka–Munk plot shown in Figure S2b. The bandgaps are 2.27, 2.36, 2.46, 2.56, and 2.67 eV for CPB, CPB-Zr-0.25, CPB-Zr-0.5, CPB-Zr-0.75, and CPB-Zr-1, respectively. Figure 2C displays the PL spectra of all samples. The CPB sample shows a PL peak centered at 521 nm, consistent with the reported values (Shamsi et al., 2016). The trend of PL result is in good agreement with that of the UV-vis diffuse reflectance spectra (Figure S3).

The TRPL spectra in Figure 2C show the lifetime of photogenerated carriers and the carrier separation kinetics. The fitting results for TRPL spectra of different samples are listed in Table S1. It is found that the carrier lifetime increases and then decreases with the increased adding amount of ZrCl_4_. Compared with the carrier lifetime (24.46 ns) of pure CPB, the CPB-Zr-0.25 shows a longer carrier lifetime at 49.38 ns, but declined gradually to 6.81 ns of CPB-Zr-1. With the small amount of ZrCl_4_ added in precursor, the Cl^−^ doped into CPB may result in the elongated carrier lifetime (Jin and Oleg, 2015). With the further increase of added ZrCl_4_, ZrO_2_ starts to form on the surface of the CPB and promotes the transfer of carriers and shortens the carrier lifetime.

The SPV spectra shown in Figure 2D, employed to evaluate the photochemical charge transfer in the catalyst, reveal that the CPB has very low photovoltage, while the photovoltage increases first with the addition of ZrCl_4_ and decreases thereafter. It is probably due to the fact that when a small amount of ZrCl_4_ is added, the surface of CPB is modified and passivated by Zr species to reduce surface defects and increase the photovoltage, while the enlarged bandgap induced by the Cl doping leads to the blue shift of the absorption edge, consistent with the UV-vis results (Zhao et al., 2016). With further increase of ZrCl_4_, the bandgap of CPB further expands and drastically reduces the absorption and leads to the decrease of surface photovoltage.

### Morphology and Microstructures Analysis

**Figure 3:**
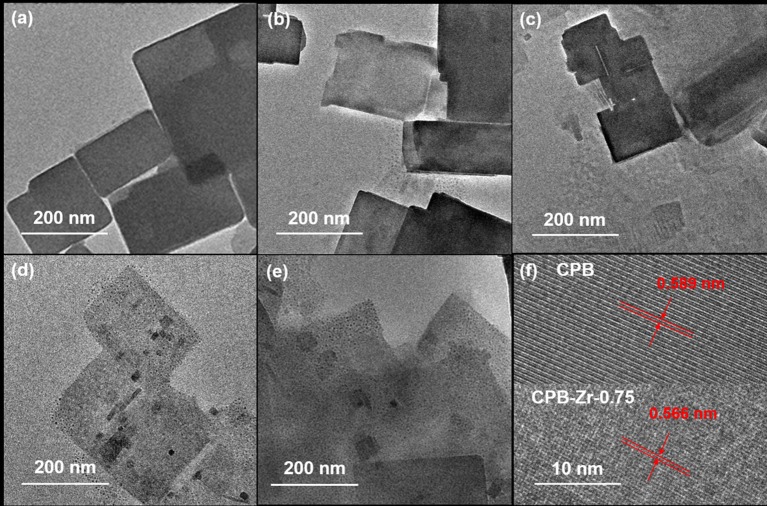
**(a–f)** TEM image of CPB and CPB-Zr-*x*: **(a)** CPB; **(b)** CPB-Zr-0.25; **(c)** CPB-Zr-0.5; **(d)** CPB-Zr-0.75; **(e)** CPB-Zr-1; **(f)** HRTEM images of CPB and CPB-Zr-0.75.

Figure 3 shows the TEM images of CPB and CPB-Zr-*x*. It can be seen that all samples consist of rectangular nanosheets with a size range of 100–400 nm. As exhibited in Figures 3d,e, small particles appear on the surface of CsPbBr_3_/Cs_4_PbBr_6_ nanosheets with the increase of ZrCl_4_ molar proportion. The high-resolution TEM in Figure 3f shows a lattice spacing of 0.589 nm determined by fast Fourier transform (FFT), which corresponds to the spacing between the (100) plane. The lattice spacing of 0.566 nm for CPB-Zr-0.75 corresponds to the distances between the (100) plane. The size of CPB-Zr-*x* nanosheet decreases with the increased amount of ZrCl_4_.

**Figure 4:**
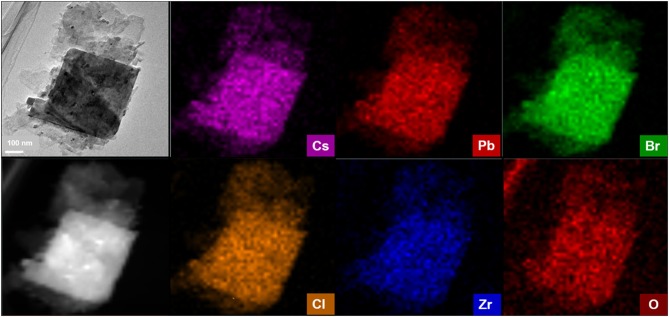
HAADF-STEM image of CPB-Zr-0.75 and the corresponding EDS elemental mapping of Cs, Pb, Br, Cl, Zr, and O in the same area.

To further study the microstructures of CPB modified by ZrCl_4_, EDX-mapping is used to map out a single nanosheet of CPB-Zr-0.75 sample under HAADF-STEM. As shown in Figure 4, the distributions of Cs, Pb, Br, Zr, Cl, and O elements can be visualized. The above elements can almost overlap in the selected region. It implies that Cl^−^ from ZrCl_4_ has been doped into CPB. As for the Zr, its distribution overlaps with the nanosheet implying that some Zr^4+^ may have been doped into the lattice while at the same time it could form ZrO_2_ nanoparticles that attach to the surface of CPB. It remains difficult to determine the exact amount of the Zr^4+^ that has been doped into the lattice. Considering that the ZrO_2_ diffraction peaks found in XRD pattern for CPB-Zr-0.75 and CPB-Zr-1, the Zr^4+^ may exist in both forms.

### Photocatalytic Performance Evaluation

**Figure 5:**
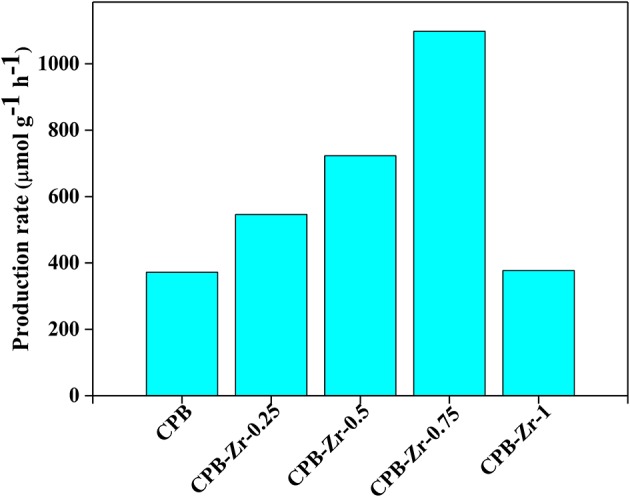
Summarized catalytic activities of CPB and CPB-Zr-*x* (*x* = 0.25, 0.5, 0.75, 1).

To evaluate the catalytic performance of the catalyst, the photocatalytic oxidation reaction of styrene was carried out to quantify their photocatalytic activity. The calculated reaction rate is summarized in Figure 5. The production rate for CPB is 372 μmol g^−1^ h^−1^. With the increase of added ZrCl_4_, it increases first and then declines, whereby the CPB-Zr-0.75 shows the maximum production rate of 1,098 μmol g^−1^ h^−1^. The comparison sample, ZrO_*x*_Cl_*y*_, prepared with the same protocol by adding ZrCl_4_ only, shows the production rate of 52 μmol g^−1^ h^−1^, suggesting that the nanosheet is critical in increasing the light absorption. One may consider that the improved photocatalytic activity originates solely from the Cl doping. To verify the role of Cl doping, we prepare the samples with the same doping level by substituting the ZrCl_4_ with CsCl for the synthesis. The CPB-Cl-*x* shows only slight improvement, with the highest production rate 1.09 times that of the pure CPB, as shown in Figure S4. It implies that both the surface functionalization of CPB with Zr species and the Cl doping are necessary in largely improving the catalytic performance toward styrene oxidation, possibly through a synergistic effect between the two methods. It also suggests that the addition of ZrCl_4_ during the synthesis for the surface modification can “kill two birds with one stone.”

It is also known that the halide perovskite is prone to be oxidized. To evaluate the structure and phase after the reaction, the catalysts are collected for structural and physicochemical analysis with XRD and PL measurements (Figure S5), showing that the XRD pattern for the catalyst after reaction is almost identical to the fresh catalyst with only a slight blue shift, indicating the chemical rigidity of the PVSK after functionalization. MAPB and MAPB-Zr-*x* (*x* = 0.25, 0.5, 0.75, 1) are prepared by the same method as above, with photocatalytic experiments shown in Figure S6. The results suggest that that the chemical modification with ZrCl_4_ can also improve the photocatalytic activity of MAPB toward the benzaldehyde production, although the baseline performance is not as good as that of CPB. The color of the MPB totally changes into white after the reaction, suggesting that the MAPB is not a robust photocatalyst as shown in Figure S7.

### UPS Spectra Analysis and Reaction Process Investigation

**Figure 6:**
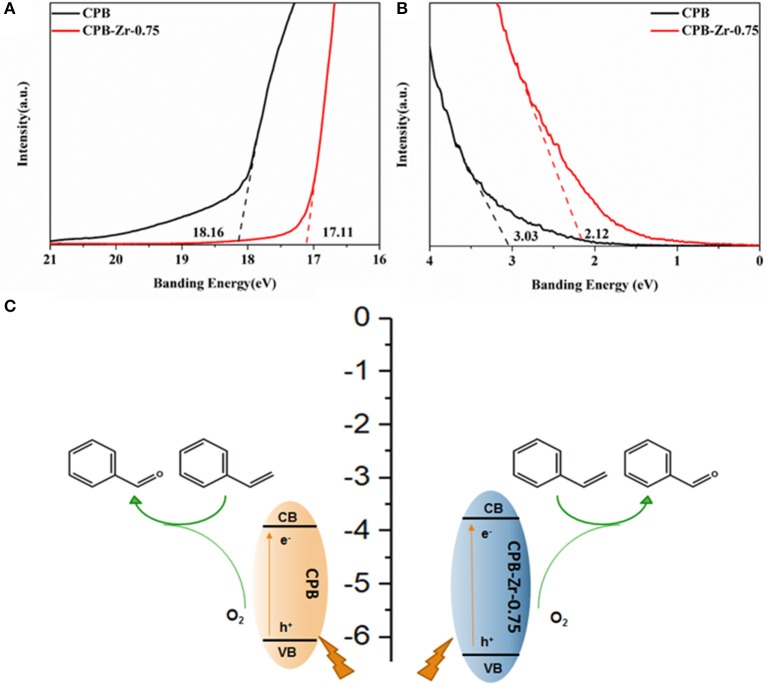
The secondary electron cutoff edge **(A)** and Fermi edge **(B)** of CPB and CPB-Zr-0.75 in UPS spectra; schematic diagram of the reaction process for oxidation of the styrene into benzaldehyde **(C)**.

To investigate the effect of ZrCl_4_ on the catalytic performance of CPB, the UPS (Figure 6) determines the valence band positions of CPB and CPB-Zr-0.75. The energy level of the CPB and CPB-Zr-0.75 is determined by using ultraviolet photoelectron spectroscopy (UPS) with a photon energy of 21.20 eV. The work function can be determined from the difference between the photon energy and the binding energy of the secondary cutoff edge (Figure 6A). Therefore, the Fermi level (E_f_) of CPB and CPB-Zr-0.75 can be determined to be −3.04 and −4.09 eV relative to the vacuum level (E_vac_), respectively. As shown in Figure 6B, the valence band (VB) spectra show that the valence band maxima (VBM) of CPB and CPB-Zr-0.75 are 3.03 and 2.12 eV below E_f_, respectively. The bandgap of CPB and CPB-Zr-0.75 are calculated by UV-vis, approximately 2.27 eV and 2.56 eV, respectively. Therefore, the energy levels of the conduction bands of the two can be calculated as −3.8 and −3.65 eV, confirming that the Cl^−^ doped into CPB may result in the widening of bandgap.

Based on the experiment, the possible reaction process is then illustrated as the scheme in Figure 6C; the visible light activates the excitons inside the CPB. Upon the separation of electron–hole pairs, the electrons transport to the surface and react with adsorbed O_2_ to generate activated oxygen species. Meanwhile, the styrene is adsorbed on the surface of the CPB and oxidized by the holes to the corresponding cationic radicals. The activated oxygen species then selectively oxidize the cationic radicals, finally leading to the formation of benzaldehyde. Due to the addition of ZrCl_4_ precursor, the chemically modified CPB can promote the generation and transfer of excitons and carriers and hence accelerates the photocatalytic production rate of benzaldehyde.

## Conclusion

In summary, we developed a surface functionalization strategy, by using ZrCl_4_ as precursor to achieve the Zr-functionalization and the Cl doping at the same time to address the carrier transport and the light absorption issue for perovskite material. For the modified CPB nanosheet, the Cl doping that widens the bandgap and the surface modification that enhances the transport of photogenerated carriers can be accomplished by simply adding ZrCl_4_ precursor in the synthesis, whereby the photocatalytic oxidation of styrene to benzaldehyde at room temperature can be largely improved. By promoting the energy transfer from CPB-Zr to O_2_, the production rate can be improved from 376 up to 1,098 μmol g^−1^ h^−1^, demonstrating the synergistic effect of Cl doping and surface modification in boosting the photocatalytic performance of CPB. In addition, this methodology can also work well for MAPbBr_3_ and hence can be employed as a general approach to enhance the photocatalytic performance of PVSK-based catalysts.

## Data Availability Statement

The raw data supporting the conclusions of this article will be made available by the authors, without undue reservation, to any qualified researcher.

## Author Contributions

PQ and QW carried out most of the experiments and wrote the manuscript. YZ, YDa, YDo, and CC carried out part of the experiments. YL and QC designed the idea and wrote the manuscript.

### Conflict of Interest

The authors declare that the research was conducted in the absence of any commercial or financial relationships that could be construed as a potential conflict of interest.
