# Supplementary material for: Fabricating Surface-Functionalized CsPbBr3/Cs4PbBr6 Nanosheets for Visible-Light Photocatalytic Oxidation of Styrene
Source: Front Chem. 2020 Mar 10;8:130. doi: 10.3389/fchem.2020.00130 (PMC7076109; doi:10.3389/fchem.2020.00130)
Supplement: Supplementary file 1 [file Data_Sheet_1.pdf]

Supplementary Material

1 Supplementary Figures and Tables

1.1 Supplementary Figures

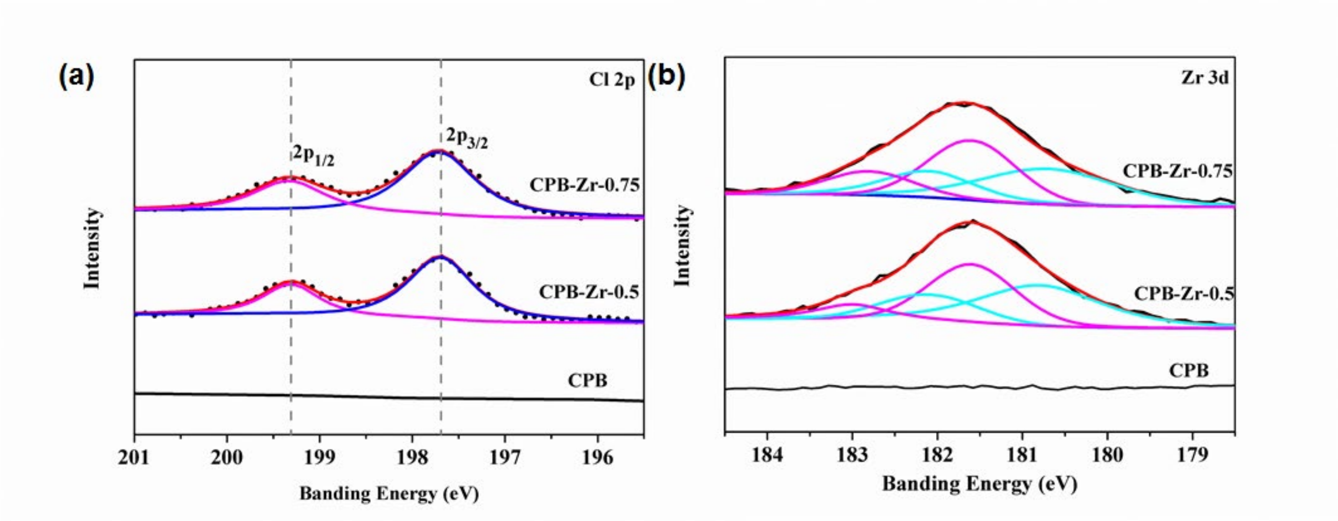

Figure S1 High-resolution XPS spectra of (a) Cl 2p, (b) Zr 3d for CPB, CPB-Zr-0.5 and CPB-Zr-0.75.

The photon energy by the simple conversion relation:

$$Energy(eV) = \frac{1240}{\lambda} \quad (1)$$

The Kubelka–Munk function is defined in terms of the following equation:

$$[F(R_\infty)\ \text{hv}]^{\frac{1}{n}} = A(h\nu - E_g) \qquad 2$$

Where $F(R_\infty)= (1-R)^2/2R$, A= proportionality constant, h= photon energy measured in eV, E_g= energy band gap and n is an index which characterizes the nature of the energy band transitions depending on whether it is direct or indirect, allowed or forbidden, *etc.* ($CsPbBr_3$ is a direct band gap type semiconductor, n= 1/2). Figure. R2 shows the Kubelka–Munk plot for all samples.

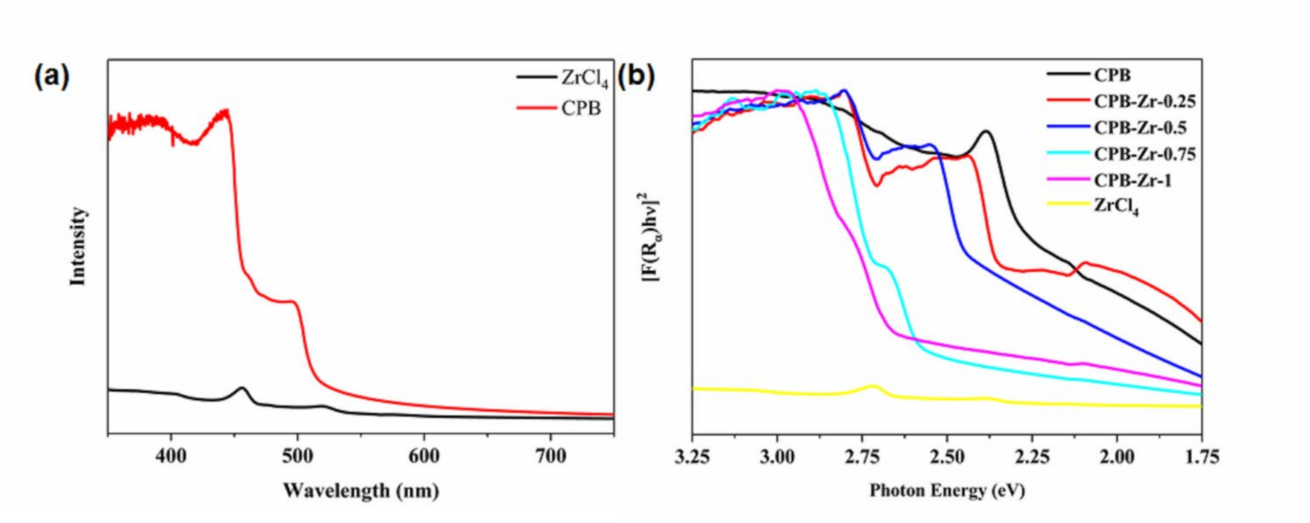

Figure S2 UV-vis diffuse reflectance spectra of $ZrCl_4$ and CPB (a). The Kubelka–Munk plot of CPB, CPB-Zr-x(x=0.25, 0.5, 0.75, 1) and $ZrCl_4$ (b).

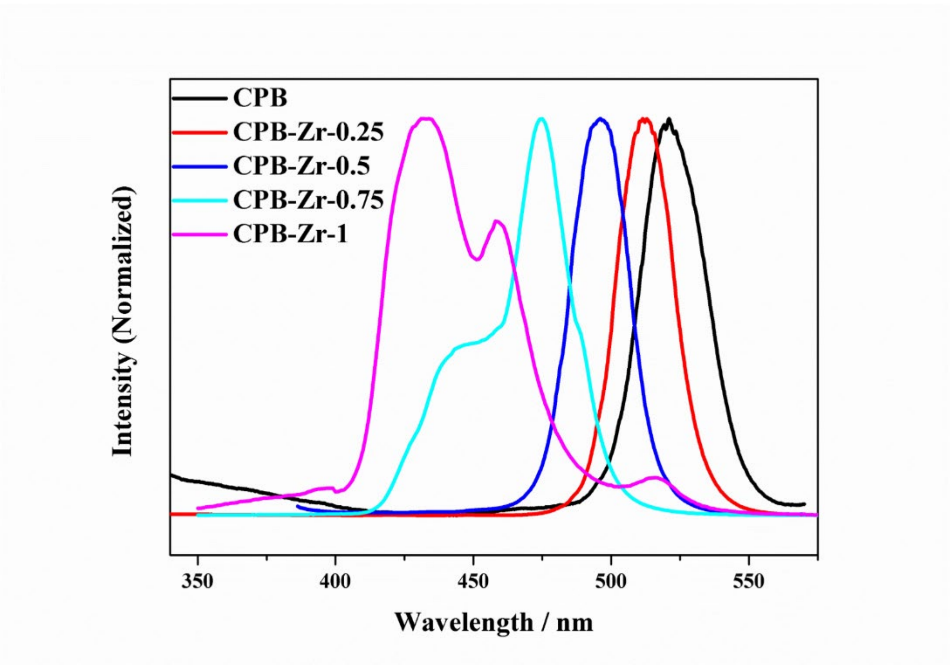

Figure S3 PL spectra of CPB and CPB-Zr-x(x=0.25, 0.5, 0.75, 1)

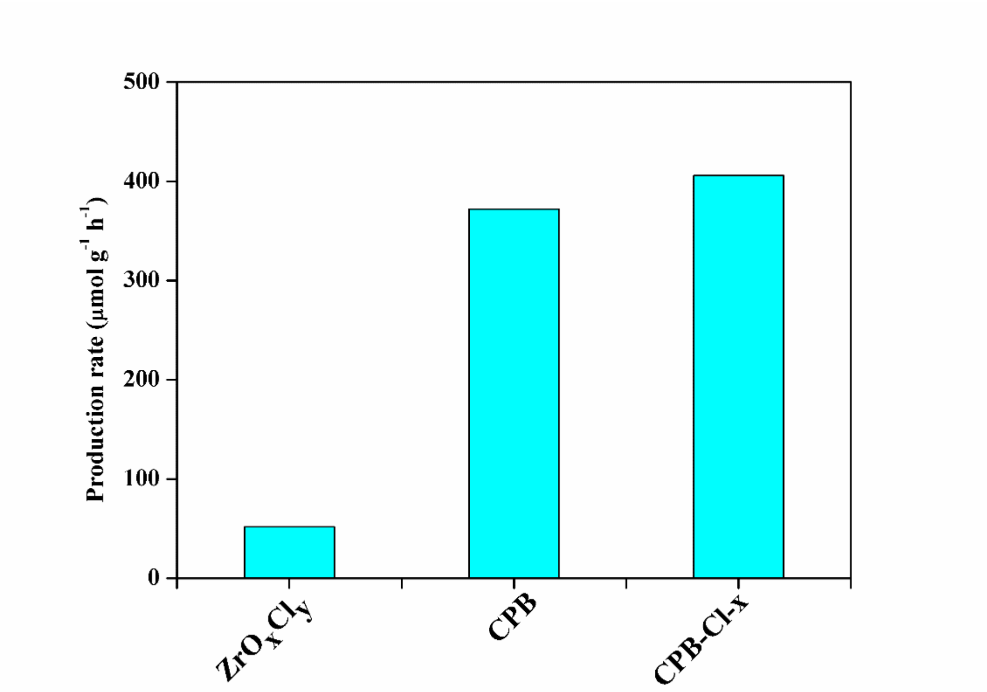

Figure S4 Catalytic activities of ZrO_xCl_y, CPB and CPB-Cl

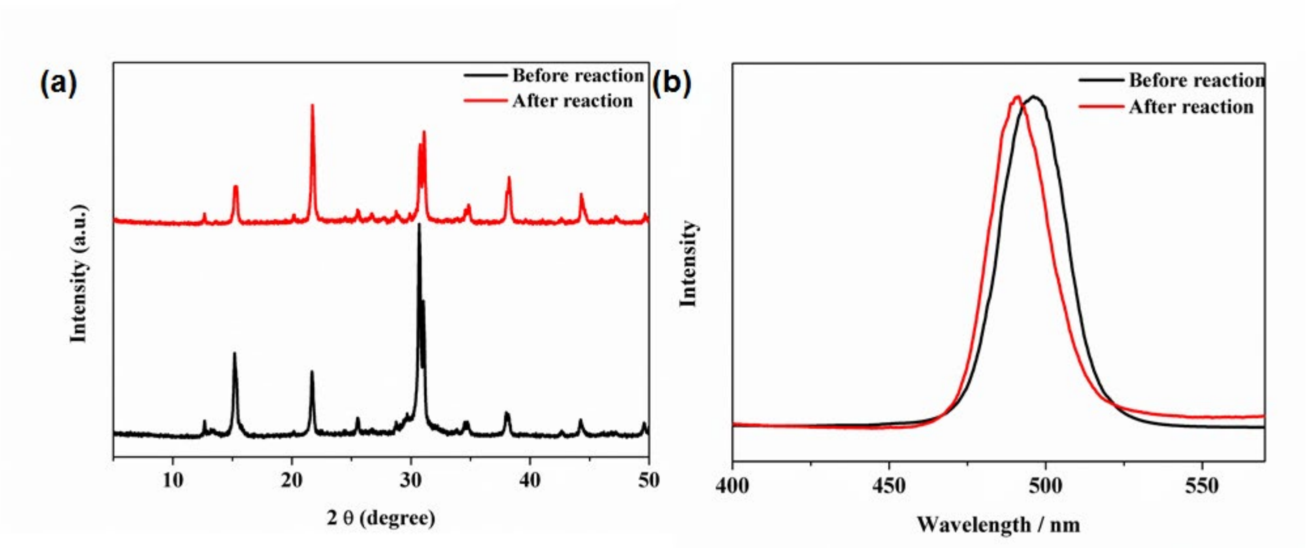

Figure S5 XRD patterns of CPB-Zr-0.75 before and after reaction (a); PL spectra of CPB-Zr-0.75 before and after reaction (b).

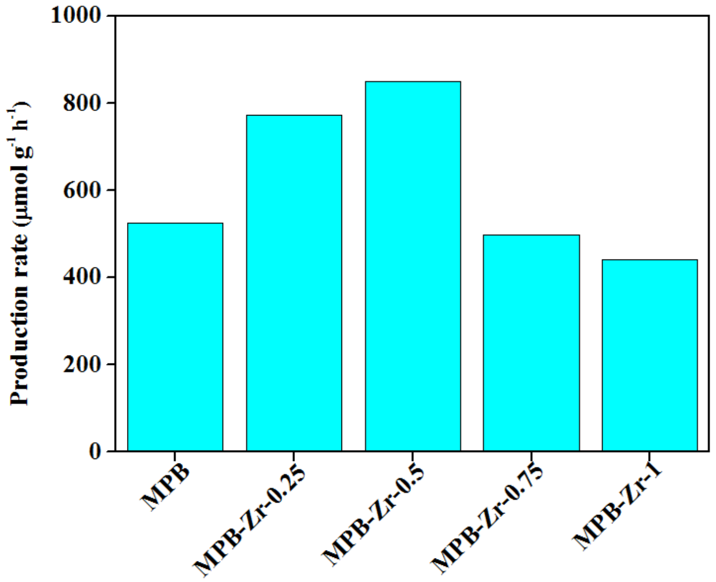

Figure S6 Summarized catalytic activities of MAPB and MAPB-Zr-x (x=0.25, 0.5, 0.75, 1).

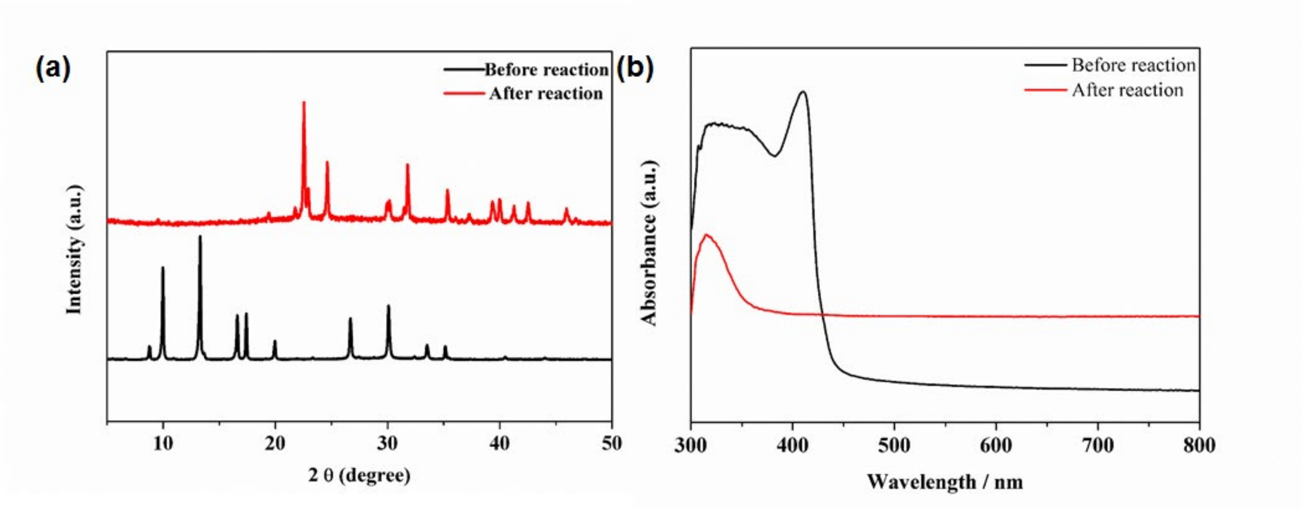

Figure S7 XRD patterns of MPB-Zr-0. 5 before and after reaction (a); UV-vis diffuse reflectance spectra of MPB-Zr-0.5 before and after reaction (b).

1.2 Supplementary Table

Table S1 The fitting results for TRPL spectra of different samples.

sample	τ_1(ns)	A_1(%)	τ_2(ns)	A_2(%)	τ_{ave}(ns)
CPB	5.735	0.408	27.639	0.498	24.46
CPB-Zr-0.25	6.802	0.220	51.562	0.566	49.38
CPB-Zr-0.5	3.512	0.309	32.900	0.504	31.09
CPB-Zr-0.75	17.771	0.374	3.642	0.540	14.54
CPB-Zr-1	9.239	0.215	1.052	0.797	6.81

Here τ indicates the exponential decay time and A represents the amplitude of exponential fitting parameter. These values are including in implemented fit equation: y = A1*exp(-x/t1) + A2*exp(-x/t2)
